# Gut microbiota and alcohol use disorder: a new frontier in treatment and recovery

**DOI:** 10.1192/bjb.2025.10129

**Published:** 2026-04

**Authors:** Valentin Skryabin

**Affiliations:** Russian Medical Academy of Continuous Professional Education, Moscow, Russia

**Keywords:** Alcohol use disorder, gut microbiota, gut–brain axis, neuroinflammation, faecal microbiota transplantation

## Abstract

**Aims and method:**

Alcohol use disorder (AUD) is a major global health concern associated with limited treatment efficacy and high relapse rates. Recent research highlights the gut microbiota as a critical modulator of AUD pathophysiology through its influence on the gut–brain axis. Chronic alcohol consumption induces gut dysbiosis, characterised by reduced microbial diversity, impaired gut barrier function and systemic inflammation, which perpetuate neuroinflammation, stress dysregulation and neurotransmitter imbalances. These disruptions exacerbate addiction-related behaviours, contributing to the cycle of dependence and relapse. This critical review synthesises current evidence on the role of gut microbiota in AUD, examining the mechanisms linking dysbiosis to addiction and evaluating therapeutic interventions such as probiotics, prebiotics, faecal microbiota transplantation (FMT), psychobiotics and dietary modifications.

**Results:**

The strategies evaluated show significant potential in restoring microbial homeostasis and improving AUD outcomes, but challenges remain, including gaps in mechanistic understanding, variability in methodologies, and barriers to clinical translation.

**Clinical implications:**

There is a need for multi-omics research, personalised medicine approaches and integrated treatment models to advance microbiota-based therapies. Gut microbiota-targeted strategies might then transform AUD management, offering innovative and personalised solutions for addiction recovery.

Alcohol use disorder (AUD) is a pervasive and multifaceted condition affecting approximately 283 million individuals globally.^1^ Characterised by compulsive alcohol consumption, inability to control intake and the development of withdrawal symptoms, AUD imposes significant burdens on individuals and society, including increased risks of liver disease, cardiovascular disorders, neuropsychiatric conditions and premature mortality. Beyond its health implications, AUD incurs substantial economic costs due to healthcare expenditure, lost productivity and legal issues.

Current treatment modalities for AUD include pharmacotherapies such as disulfiram (an aldehyde dehydrogenase inhibitor), naltrexone (a μ-opioid receptor antagonist) and acamprosate (a glutamate modulator), alongside behavioural interventions like cognitive–behavioural therapy (CBT).^2^ Despite their effectiveness in some individuals, these approaches fail to address the complex neurobiological and systemic factors underlying addiction, contributing to high relapse rates, especially within the first year of treatment.^3^ Such limitations underscore the urgent need for novel, integrative treatment approaches.

Recent research has shifted attention towards the gut microbiota – a diverse microbial ecosystem within the gastrointestinal tract – as a significant player in the pathophysiology of AUD. The gut microbiota contributes to host health by regulating immune function, metabolism and brain activity through bidirectional communication pathways termed the gut–brain axis.^4^ Chronic alcohol consumption disrupts gut microbial diversity, leading to dysbiosis characterised by overgrowth of pathogenic taxa, impaired intestinal barrier integrity and increased systemic inflammation. These changes exacerbate neuroinflammatory processes, impair neurotransmitter signalling and dysregulate stress response systems, perpetuating alcohol-seeking behaviours.^5^

Although prior reviews have broadly discussed the gut–brain axis in AUD,^6,7^ they have not sufficiently explored empirical research and mechanistic pathways in depth. This review aims to provide a critical synthesis of original studies, elucidating the mechanisms linking gut dysbiosis to AUD and evaluating microbiota-targeted interventions. It also critically assesses methodological limitations in faecal microbiota transplantation (FMT) trials, including donor–recipient compatibility and long-term safety. By synthesising findings from preclinical and clinical studies, this review aims to establish the gut microbiota as a promising target for novel interventions in addiction treatment.

## Method

We conducted a focused narrative review using PubMed and Scopus databases, covering studies published between 2000 and 2024. Included studies were peer-reviewed original articles (2017–2024) investigating gut microbiota in AUD, with mechanistic or clinical relevance. Reviews were identified only for contextual framing. The search terms were: ‘alcohol use disorder’, ‘gut microbiota’, ‘FMT’, ‘psychobiotics’. Non-English-language articles and studies without control groups were excluded.

## Results

### Gut dysbiosis in AUD: pathophysiological insights

Chronic alcohol consumption induces profound alterations in the composition and function of gut microbiota, a condition known as dysbiosis. Dysbiosis is characterised by reduced microbial diversity (e.g. depletion of *Faecalibacterium prausnitzii* and *Roseburia* spp.), impaired gut barrier integrity and overgrowth of endotoxin-producing *Proteobacteria*. Studies consistently report that individuals with AUD exhibit significant reductions in beneficial bacteria such as *Lactobacillus* and *Bifidobacterium* and an overgrowth of potentially harmful species, including *Proteobacteria* and *Enterobacteriaceae*.^6,7^ Leclercq et al^8^ found that these alterations were associated with elevated levels of lipopolysaccharides (LPS) and increased systemic inflammation, directly linking dysbiosis to neuroimmune activation in AUD. Ferrere et al^9^ demonstrated in a murine model that ethanol exposure increases intestinal permeability via downregulation of tight junction proteins (occludin, zonula occludens-1), facilitating LPS translocation. Similarly, Segovia-Rodriguez et al^10^ demonstrated that alcohol-dependent individuals exhibited microbiota profiles distinct from healthy controls, with reduced *Faecalibacterium* and *Roseburia*, key producers of anti-inflammatory short-chain fatty acids (SCFAs).

This imbalance compromises gut homeostasis, impairing the protective functions of the gut barrier. Alcohol exacerbates this disruption by increasing gut permeability, a phenomenon often referred to as ‘leaky gut’. The weakened intestinal barrier allows bacterial endotoxins such as LPS to translocate into systemic circulation, triggering systemic and neuroinflammatory responses.^5^ Elevated levels of circulating LPS are a hallmark of alcohol-related dysbiosis and have been directly linked to the activation of immune pathways, which perpetuate the inflammatory state associated with AUD.

In addition to barrier dysfunction, alcohol-induced dysbiosis alters the metabolic activity of gut microbiota, particularly the production of SCFAs such as butyrate, acetate and propionate. SCFAs are essential metabolites that regulate gut–brain communication, immune modulation and energy metabolism.^6^ Decreased SCFA levels in people with AUD contribute to systemic inflammation and impair neuroprotective mechanisms, further exacerbating the cycle of addiction and its associated cognitive and behavioural deficits.

Interestingly, the impact of dysbiosis is not confined to inflammatory pathways but extends to the modulation of gut–brain signalling. Gut microbiota interact with the enteric nervous system (ENS), a dense network of neurons embedded in the gastrointestinal tract, to influence central nervous system (CNS) function. Dysbiosis disrupts this communication, affecting the synthesis of key neurotransmitters such as serotonin and dopamine, which are crucial for mood regulation and reward processing.^11,12^ These disruptions may explain the heightened levels of anxiety, depression and compulsive alcohol-seeking behaviour observed in individuals with AUD.

Gut microbiota modulate serotonin synthesis through species-specific pathways. For example, Yu et al^13^ demonstrated in gnotobiotic mice that *Turicibacter sanguinis* converts dietary tryptophan to 5-hydroxytryptamine (5-HT) via the *tnaA* gene. Although direct measurements of faecal serotonin in people with AUD are limited, dysbiosis in AUD is associated with reduced tryptophan availability, a precursor for 5-HT synthesis, as observed in serum metabolomic studies.^14^ This depletion may contribute to mood disturbances and reward system dysfunction.

Gamma-aminobutyric acid (GABA) synthesis is mediated by *Lactobacillus rhamnosus* (JB-1), which utilises glutamate decarboxylase to generate GABA, a key inhibitory neurotransmitter. In ethanol-exposed rodent models, administration of JB-1 reduced anxiety-like behaviours, supporting the role of gut-derived GABA in AUD-related stress modulation.^15^

Dopamine metabolism is also influenced by microbial activity. *Bacteroides thetaiotaomicron* produces phenyllactic acid, a precursor for dopamine synthesis in the striatum, a region critically involved in reward processing and addiction.^16^ Altered dopamine signalling due to gut microbiota dysbiosis may contribute to anhedonia and compulsive alcohol-seeking behaviours in people with AUD. By disrupting these neurotransmitter pathways, dysbiosis perpetuates the neurobiological and behavioural components of alcohol addiction, reinforcing the cycle of dependence and relapse.

Preclinical studies have provided critical insights into the causal relationship between dysbiosis and AUD-related behaviours. In murine models, alcohol exposure consistently led to reductions in microbial diversity and increased intestinal permeability. When dysbiotic microbiota from alcohol-exposed mice were transplanted into germ-free mice, the recipients exhibited heightened anxiety-like behaviours and increased alcohol consumption compared with controls.^17^ These findings suggest that dysbiosis is not merely a consequence of alcohol consumption but an active contributor to AUD pathophysiology.

Moreover, emerging research highlights that the extent of gut dysbiosis correlates with the severity of AUD. A study by Leclercq et al^18^ found that people with more severe alcohol dependence exhibited greater reductions in microbial diversity and higher levels of circulating LPS. This dose-dependent relationship underscores the importance of addressing gut dysbiosis in the management of AUD. Notably, early intervention to restore microbial balance may mitigate the progression of addiction-related behaviours and improve treatment outcomes.

**Fig. 1:**
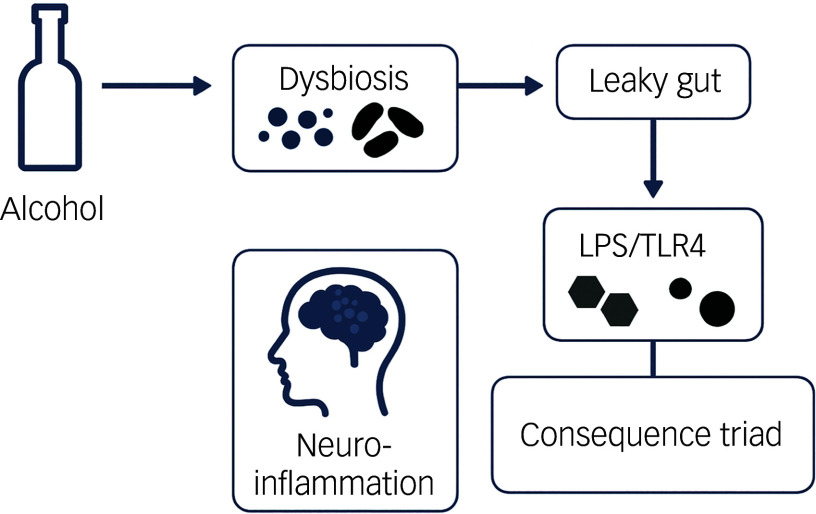
Pathophysiological pathways in alcohol use disorder. Alcohol consumption induces microbial dysbiosis (blue denotes beneficial, black denotes pathogenic). Dysbiosis compromises intestinal barrier integrity, permitting translocation of lipopolysaccharides (LPS) into systemic circulation (‘leaky gut’). LPS activate Toll-like receptor 4 (TLR4) pathways, triggering neuroinflammatory cascades and a triad of consequences: disruption of reward processing, hypothalamic–pituitary–adrenal (HPA) axis regulation and neurotransmitter balance.

In conclusion, gut dysbiosis is a critical and modifiable factor in AUD pathophysiology. By disrupting the gut barrier, altering microbial metabolite production and impairing gut–brain communication, dysbiosis exacerbates the neurobiological and behavioural aspects of addiction. These findings highlight the potential of targeting the gut microbiota as a novel therapeutic strategy for AUD, a concept explored further in the therapeutic implications section of this review. Figure 1 summarises the key pathophysiological pathways linking gut dysbiosis to AUD progression, including microbial shifts, barrier dysfunction and downstream neurobehavioral effects.

Table 1 synthesises key preclinical and clinical studies demonstrating causal links between gut dysbiosis and AUD progression.

**Table 1:** Key preclinical and clinical studies linking gut dysbiosis to pathophysiology in alcohol use disorder (AUD)

Study (year)	Model (preclinical/ clinical)	Key microbiota alterations	Functional outcomes
Leclercq et al (2020)^8^	Clinical	↓ *Faecalibacterium* ↑ *Proteobacteria*	↑ LPS ↑ Neuroinflammation ↓ Sociability
Ferrere et al (2017)^9^	Preclinical (mice)	↑ Pathobionts ↓ Tight junctions	↑ Gut permeability ↑ Liver inflammation
Segovia-Rodriguez et al (2022)^10^	Clinical	↓ SCFA producers	↑ Alcohol craving ↓ Cognitive function
Zhao et al (2020)^17^	Preclinical (mice)	FMT from AUD → anxiety	↓ mGluR1 ↑ Depression-like behaviour
Bajaj et al (2021)^34^	Clinical (RCT)	FMT→ temporary ↑ in microbial diversity	↓ Short-term craving, no long-term benefit

LPS, lipopolysaccharides; SCFA, short-chain fatty acid; RCT, randomised controlled trial; FMT, faecal microbiota transplantation; mGluR1, metabotropic glutamate receptor 1.

### Mechanistic pathways linking gut dysbiosis to AUD

#### Neuroinflammation

Neuroinflammation is a key mediator in the pathophysiology of AUD, and the gut microbiota plays a central role in its regulation. Chronic alcohol consumption triggers neuroinflammation through multiple pathways. Ethanol metabolites (e.g. acetaldehyde) promote NOD-, LRR- and pyrin-domain-containing protein 3 (NLRP3) inflammasome signalling in microglia, as shown in post-mortem human brain tissue.^19^ Peripheral cytokine infiltration, particularly tumour necrosis factor alpha (TNF-α) and interleukin 6 (IL-6), disrupt blood–brain barrier integrity via matrix metalloproteinase-9 (MMP-9) activation, allowing cytokine entry into the CNS.^20^ Additionally, gut-derived LPS activate the vagus nerve, which transmits pro-inflammatory signals to the nucleus tractus solitarius, further amplifying neuroinflammatory cascades.^21^ These interconnected pathways contribute to the sustained neuroimmune dysregulation observed in people with AUD, reinforcing alcohol-seeking behaviours.

Chronic alcohol consumption disrupts the gut barrier, resulting in increased intestinal permeability. This dysfunction allows bacterial endotoxins to enter systemic circulation. LPS activates Toll-like receptor 4 (TLR4) pathways, triggering the release of pro-inflammatory cytokines, including TNF-α and IL-6.^5^ These cytokines cross the blood–brain barrier, where they activate microglia, the brain’s resident immune cells.

Once activated, microglia perpetuate neuroinflammation by producing additional inflammatory mediators that disrupt neural plasticity and impair synaptic function. This inflammatory environment contributes to alterations in reward circuitry within the mesolimbic pathway, particularly in areas such as the ventral tegmental area (VTA) and nucleus accumbens. Dysregulated reward signalling reinforces alcohol-seeking behaviour, creating a feedback loop that exacerbates addiction.^7^ Furthermore, neuroinflammation has been implicated in cognitive deficits and mood disorders, both of which are common comorbidities in AUD.

Animal studies provide compelling evidence for the link between gut dysbiosis and neuroinflammation. In murine models, alcohol-induced dysbiosis was associated with elevated systemic LPS levels and increased activation of brain microglia.^6^ Interventions such as faecal microbiota transplantation (FMT) from healthy donors reversed these effects, reducing neuroinflammatory markers and improving cognitive outcomes. Translating these findings into human studies is a critical step in validating the therapeutic potential of targeting neuroinflammation through gut microbiota modulation.

#### Stress axis dysregulation

The hypothalamic–pituitary–adrenal (HPA) axis, a central regulator of the body’s stress response, is intimately connected to gut–brain communication. Dysbiosis induced by chronic alcohol consumption has been shown to exaggerate HPA axis activity, leading to heightened cortisol levels and increased stress reactivity.^22^ This dysregulation creates a vulnerability to stress-induced alcohol consumption, as individuals often turn to alcohol as a maladaptive coping mechanism.

The bidirectional communication between the gut and the HPA axis involves multiple pathways. Gut microbiota influence the production of stress-modulating molecules, such as corticotropin-releasing hormone (CRH), which initiates the HPA axis cascade. Dysbiosis alters this balance, resulting in excessive CRH release and sustained HPA axis activation. Elevated cortisol levels, in turn, further disrupt gut microbiota composition, creating a vicious cycle that perpetuates dysbiosis and stress axis dysregulation.^23^

Preclinical studies have demonstrated the impact of gut microbiota on stress responses. In germ-free mice, absence of gut microbiota was associated with exaggerated HPA axis activation in response to stress. Restoring microbiota through probiotic supplementation or FMT normalised HPA axis activity, reducing anxiety-like behaviours and alcohol preference.^24^ These findings suggest that modulating gut microbiota could serve as a therapeutic strategy for mitigating stress-related triggers in AUD.

#### Neurotransmitter imbalances

The gut microbiota plays a pivotal role in the synthesis and regulation of neurotransmitters that influence brain function and behaviour. For example, gut bacteria are involved in producing GABA, serotonin, dopamine and noradrenaline, all of which are critical in regulating mood, cognition and reward processing.^14^ Chronic alcohol consumption disrupts microbial populations responsible for these processes, contributing to neurotransmitter imbalances that exacerbate addiction behaviours.

One of the most well-studied examples is serotonin. Approximately 90% of the body’s serotonin is synthesised in the gut, with gut bacteria such as *Lactobacillus* and *Bifidobacterium* playing essential roles in its production.^25^ Dysbiosis reduces serotonin availability, which is associated with depressive symptoms commonly observed in individuals with AUD. Depressive symptoms, in turn, increase the risk of relapse, further perpetuating the cycle of addiction.

Similarly, dopamine, a key neurotransmitter in the brain’s reward system, is influenced by gut microbial metabolites such as SCFAs. Dysbiosis impairs dopamine signalling, leading to anhedonia (reduced ability to experience pleasure) and increased alcohol-seeking behaviour as a compensatory mechanism. Studies in rodent models have shown that restoring gut microbial balance through probiotics can enhance dopamine signalling, reducing alcohol intake and improving motivation.^26^

Finally, GABA, the primary inhibitory neurotransmitter in the brain, is modulated by gut microbiota through pathways involving specific bacterial strains. Alcohol disrupts these pathways, contributing to heightened anxiety and irritability during withdrawal. Probiotic interventions targeting GABA production have shown promise in reducing these withdrawal symptoms, highlighting the therapeutic potential of restoring gut–brain neurotransmitter balance in AUD.^15^

### Therapeutic interventions

Advances in our understanding of the gut microbiota and its role in AUD have opened new avenues for therapeutic interventions. Strategies aimed at restoring microbial balance and enhancing gut–brain communication hold significant promise in mitigating AUD pathophysiology. Key approaches include the use of probiotics, prebiotics, FMT, psychobiotics and dietary interventions. Each of these modalities offers unique mechanisms for targeting the disruptions caused by alcohol-induced dysbiosis.

#### Probiotics and prebiotics

Probiotics, which are live microorganisms that confer health benefits when consumed in adequate amounts, represent one of the most extensively studied therapeutic options for AUD. Specific strains such as *Lactobacillus rhamnosus* and *Bifidobacterium bifidum* have demonstrated efficacy in preclinical studies, where they were shown to restore gut barrier function, reduce systemic inflammation and modulate neuroinflammatory responses.^27,28^ Probiotics modulate gut barrier function, potentially reducing systemic inflammation and alcohol-induced neurotoxicity. For instance, Zhang et al^29^ reported that *Lactobacillus casei* supplementation reduced serum zonulin (a marker of gut permeability) by 25% in a 12-week randomised controlled trial (RCT). However, the extent of probiotics’ impact remains debated.

Prebiotics, non-digestible fibres that promote the growth of beneficial microbiota, complement the effects of probiotics. Compounds such as inulin and fructo-oligosaccharides have been shown to increase SCFA production, particularly butyrate, which exerts anti-inflammatory effects and supports gut barrier integrity.^30^ In preclinical models, prebiotic supplementation reversed alcohol-induced dysbiosis and reduced alcohol-seeking behaviour.^31^ Combining probiotics with prebiotics into symbiotic formulations offers a synergistic approach, enhancing microbial diversity and metabolic activity while addressing the multifaceted disruptions caused by AUD.

Despite these promising findings, challenges remain. Variability in probiotic strains and dosages across studies limits the generalisability of results, and long-term clinical trials are needed to establish their efficacy in diverse patient populations. Moreover, adherence to probiotic regimens, particularly in the context of AUD-related cognitive and motivational impairments, poses a practical challenge to widespread implementation.

#### Faecal microbiota transplantation (FMT)

Faecal microbiota transplantation (FMT) involves the transfer of gut microbiota from a healthy donor to a recipient, with the aim of restoring microbial diversity and functionality.^32^ Although FMT is well-established in treating conditions such as *Clostridioides difficile* infection, its application in AUD is still in its early stages. Preclinical studies have provided compelling evidence for the efficacy of FMT in reversing alcohol-induced dysbiosis and reducing addiction-related behaviours. In murine models, FMT from healthy donors restored gut barrier integrity, normalised microbial composition and attenuated alcohol-induced neuroinflammation, resulting in decreased alcohol consumption.^6^

The therapeutic potential of FMT extends beyond its ability to address dysbiosis. By introducing a diverse microbial community, FMT may also enhance the production of SCFAs and neurotransmitter precursors, thereby improving gut–brain communication. In addition, FMT has been shown to modulate immune responses, reducing the pro-inflammatory state associated with chronic alcohol use. Ezquer et al^33^ reported that FMT from healthy donors reduced LPS levels, restored gut microbiota diversity and decreased alcohol-seeking behaviour in preclinical models. Leclercq et al^8^ demonstrated in a preclinical murine AUD model that FMT from healthy donors reduced hippocampal TNF-α levels by 60% and restored spatial memory in the Morris water maze.^8^ However, Bajaj et al^34^ reported mixed results in a human phase 1, double-blind RCT: although FMT was found to be safe and associated with short-term reduction in alcohol craving and consumption with favourable microbial changes versus placebo in individuals with alcohol-associated cirrhosis, microbial diversity reverted to baseline after 12 weeks, highlighting durability challenges. However, these findings remain preliminary and require replication in larger clinical cohorts.

Despite its promise, FMT faces several challenges in clinical translation. Safety concerns related to the potential transmission of pathogens and variability in donor microbiota composition necessitate stringent donor screening and standardised protocols.^35,36^ Ethical considerations, particularly regarding informed consent and equitable access, must also be addressed. Furthermore, public perception of FMT as an invasive or unappealing procedure may limit patient acceptance, highlighting the need for effective communication and education.

To advance FMT as a treatment for AUD, rigorous clinical trials are required to establish its efficacy and safety in human populations. Longitudinal studies investigating the durability of its effects and its impact on relapse rates are particularly important. As research progresses, encapsulated forms of FMT, which offer a less invasive alternative to traditional procedures, may enhance its feasibility and acceptance in clinical practice.

#### Psychobiotics

Psychobiotics, a subclass of probiotics with specific effects on mental health, have garnered attention as a potential therapeutic strategy for AUD. These agents influence the gut–brain axis by modulating neurotransmitter production, reducing systemic inflammation and neuroinflammation, and normalising HPA axis activity.^37^ For instance, strains such as *Bifidobacterium longum* and *Lactobacillus helveticus* have been shown to alleviate anxiety and depressive symptoms in preclinical models, both of which are common comorbidities in AUD.^38^

In the context of addiction, psychobiotics may offer dual benefits by addressing the psychological and physiological dimensions of AUD. By enhancing the production of serotonin and GABA, psychobiotics can regulate mood and reduce stress reactivity, which are critical factors in relapse prevention.^39^ Additionally, their anti-inflammatory properties may mitigate neuroinflammation, thereby improving cognitive function and reward processing.

The mechanistic basis of psychobiotics extends beyond AUD to other psychiatric disorders, such as depression and schizophrenia, where gut dysbiosis similarly disrupts neurotransmitter balance (e.g. serotonin, GABA), gut barrier integrity and neuroimmune signalling.^7,11,12,14^ Critically, conventional psychotropic medications (e.g. antidepressants, antipsychotics) may themselves induce dysbiosis or alter microbial metabolism,^14,37^ creating a therapeutic paradox. This underscores the need for integrated approaches that consider microbiota–medication interactions – particularly in AUD, where comorbidities often necessitate polypharmacy.

Preliminary clinical studies investigating psychobiotics in AUD populations have yielded promising results. Patients receiving psychobiotic supplementation reported improvements in emotional well-being, reduced alcohol cravings and better adherence to abstinence protocols. Although there was a general agreement on the effectiveness of psychobiotics, the variability in treatment approaches and clinical presentations limits the comparability and generalisation of the findings.^40^ Larger-scale trials are needed to validate these findings and identify the specific strains and dosages that are most effective.

The integration of psychobiotics into AUD treatment frameworks offers a unique opportunity to address the gut–brain axis as a therapeutic target. Future research should explore the combined use of psychobiotics with behavioural and pharmacological therapies to maximise their efficacy and ensure comprehensive care.

#### Dietary interventions

Dietary interventions are a practical and non-invasive approach to improving gut health in AUD populations. Diets rich in fibre, fermented foods and polyphenols have been shown to enhance microbial diversity, support gut barrier function and reduce systemic inflammation.^41^ For example, a high-fibre diet promotes the growth of beneficial bacteria that produce SCFAs, which play a critical role in maintaining gut–brain communication.

Fermented foods, such as yogurt, kefir and kimchi, naturally contain probiotics that can supplement microbial diversity. Polyphenol-rich foods, including berries, tea and dark chocolate, exhibit anti-inflammatory and antioxidative properties that support gut and brain health. These dietary components may counteract the detrimental effects of alcohol-induced dysbiosis, thereby improving overall health outcomes.

Integrating dietary counselling into AUD treatment programmes could provide a cost-effective and accessible adjunct to existing therapies. By empowering patients with knowledge about the impact of diet on gut health, clinicians can encourage sustainable lifestyle changes that support recovery. However, dietary interventions should be tailored to individual needs and preferences, particularly in populations with coexisting conditions or dietary restrictions.

Further research is needed to establish evidence-based dietary guidelines for people with AUD, with a focus on optimising microbial diversity and function. Longitudinal studies examining the impact of dietary interventions on relapse rates and psychological outcomes would provide valuable insights into their long-term efficacy.

## Discussion

Alcohol use disorder is a complex and multifaceted condition with profound individual and societal impacts. As shown in this review, emerging evidence positions the gut microbiota as a critical player in AUD pathophysiology, influencing neuroinflammation, stress responses and neurotransmitter regulation through the gut-brain axis. Chronic alcohol consumption disrupts gut microbial balance, leading to dysbiosis, characterised by reduced microbial diversity, gut barrier dysfunction and systemic inflammation. These disruptions exacerbate addiction-related behaviours, reinforcing the cycle of dependence and relapse.

Therapeutic interventions targeting the gut microbiota, including probiotics, prebiotics, FMT, psychobiotics and dietary modifications, show significant promise in restoring microbial homeostasis and alleviating AUD symptoms, but several challenges must be addressed to translate these findings into effective clinical interventions. These include gaps in understanding the mechanisms underlying gut–brain interactions, variability in research methodologies, barriers to clinical translation and the ethical considerations surrounding microbiota-based therapies. This section explores these challenges in detail and proposes actionable strategies to overcome them.

### Gaps in mechanistic understanding

Despite significant advances in understanding the role of gut microbiota in AUD, the precise mechanisms linking dysbiosis to addiction-related behaviours remain poorly understood. For example, although it is known that microbial metabolites such as SCFAs influence brain function, the specific pathways by which they modulate reward circuits or stress responses are not fully elucidated. Similarly, the interplay between gut-derived inflammatory signals and central neuroinflammatory processes requires further investigation.

One of the most critical gaps is the lack of comprehensive multi-omics studies that integrate metagenomics, metabolomics and transcriptomics to capture the complex interactions between gut microbiota and host systems. These approaches could identify key microbial species, metabolites and host genes involved in AUD pathophysiology. For instance, studies could explore how dysbiosis-induced changes in SCFA levels affect neurotransmitter synthesis or how microbial endotoxins alter microglial activity in specific brain regions.

Another area needing clarification is the temporal relationship between dysbiosis and progression of AUD. Longitudinal studies tracking microbiota changes from early alcohol use through chronic dependence and recovery phases could provide valuable insights into causal relationships and critical intervention windows. Understanding these dynamics is essential for designing therapies that address the root causes of addiction rather than its symptoms.

### Variability in research methodologies

The lack of standardisation in microbiota research presents a significant barrier to advancing the field. Studies investigating the gut microbiota in AUD often differ in their methodologies, including sample collection techniques, sequencing platforms and data analysis pipelines. These inconsistencies make it challenging to compare results across studies or draw definitive conclusions about the role of specific microbial species or metabolites.

For example, variations in the use of 16S rRNA sequencing versus whole-genome metagenomics can lead to different levels of taxonomic resolution, affecting the identification of microbial changes associated with AUD. Furthermore, differences in how dysbiosis is quantified – such as microbial diversity indices versus functional gene profiling – contribute to heterogeneity in findings. Establishing standardised protocols for sample collection, microbial profiling and data interpretation is crucial for ensuring reproducibility and facilitating meta-analyses.

Additionally, most studies focus on single time points, providing only a snapshot of microbial changes. Longitudinal designs that assess microbiota dynamics over time are needed to capture the fluctuating nature of gut ecosystems, particularly in response to interventions such as probiotics or FMT. By addressing these methodological issues, researchers can build a more robust evidence base to guide clinical applications.

### Barriers to clinical translation

Translating microbiota-targeted therapies from research settings to clinical practice involves several logistical, safety and feasibility challenges. One of the primary concerns is the long-term safety of interventions such as probiotics and FMT. Although these therapies have shown promise in preclinical and pilot studies, their effects on human microbiota over extended periods are not well understood. For example, the introduction of exogenous microbial strains through probiotics or FMT could lead to unintended alterations in the host microbiome, potentially resulting in adverse effects.

Another barrier is the scalability of these interventions. Probiotic supplementation often requires consistent, long-term adherence, which may be difficult for individuals with AUD, especially those experiencing cognitive impairments or socioeconomic constraints. Similarly, the cost and logistical complexity of FMT, including donor screening and preparation, limit its accessibility in clinical settings.

Acceptance by patients also poses a challenge. Interventions like FMT, which involve the transfer of faecal material, may be perceived as invasive or unappealing, reducing their uptake. Addressing these barriers will require not only technical advancements, such as encapsulated forms of FMT, but also effective communication strategies to educate patients and reduce stigma associated with microbiota-based therapies.

### Ethical and regulatory considerations

The use of microbiota-based interventions raises important ethical and regulatory questions. For instance, donor selection for FMT involves rigorous screening to minimise the risk of pathogen transmission. However, the lack of universally accepted guidelines for donor screening and microbiota preparation creates variability in safety standards across studies and institutions. Developing global regulatory frameworks that ensure consistency and transparency is essential for advancing FMT as a mainstream therapy.

Equitable access to microbiota-targeted interventions is another ethical concern. Probiotic supplements and FMT procedures may be cost-prohibitive for underserved populations, exacerbating health disparities in AUD treatment. Policymakers must consider strategies to subsidise these therapies or integrate them into public health initiatives to ensure their availability to all individuals, regardless of socioeconomic status.

Additionally, the potential commercialisation of microbiota-based therapies raises questions about intellectual property and profit-driven practices. For example, proprietary probiotic formulations could limit accessibility, and over-the-counter products with unproven efficacy could mislead consumers. Establishing regulatory oversight to differentiate evidence-based therapies from unsubstantiated claims will be critical for maintaining public trust and maximising therapeutic benefits.

### Future directions

Future research on microbiota-targeted therapies for AUD must prioritise uncovering the precise mechanisms linking gut dysbiosis to addiction-related behaviours. Multi-omic approaches, integrating metagenomics, metabolomic, and transcriptomics, will be instrumental in identifying key microbial species and metabolites involved in neuroinflammatory processes, stress axis dysregulation and neurotransmitter imbalances. Longitudinal studies tracking microbiota changes throughout the progression and recovery of AUD are equally critical to establish temporal relationships and identify optimal intervention windows.

Personalised medicine is a promising frontier, with advances in microbiota profiling enabling the development of tailored interventions. Precision therapies could leverage individual microbial compositions to predict responses to probiotics, prebiotics or FMT. By integrating microbiota data with genetic, dietary and psychosocial factors, clinicians can design targeted strategies to enhance treatment efficacy and reduce relapse rates, paving the way for a more holistic approach to addiction management.

Integrated treatment models combining microbiota-focused therapies with existing pharmacological and behavioural interventions hold significant potential. For example, psychobiotics targeting mood regulation could complement CBT, while dietary modifications could synergise with medications like naltrexone. Expanding public health initiatives to include education on gut health and accessible dietary interventions will further support recovery efforts, reducing health disparities and fostering sustainable lifestyle changes.

Finally, large-scale, standardised clinical trials are essential to validate the efficacy and safety of microbiota-targeted therapies. These trials should include diverse populations and robust biomarkers to evaluate outcomes such as relapse prevention, emotional well-being and cognitive recovery. Addressing these research priorities will be pivotal in translating microbiota science into actionable solutions for AUD, offering new hope in addiction medicine.

Although clinical application of microbiota-targeted therapies in AUD remains in its early stages, accumulating preclinical and early clinical evidence supports their promise. With advances in precision microbiome profiling, psychobiotic development and non-invasive delivery methods (e.g. oral FMT capsules), translation into clinical practice is increasingly feasible. Moreover, understanding gut–brain mechanisms may offer novel biomarkers of relapse vulnerability and inform adjunctive strategies alongside existing treatments.

## Data Availability

The data that support the findings of this study are available from the corresponding author upon reasonable request.

## About the author

**Valentin Skryabin**, Associate Professor, Department of Addiction Psychiatry, Russian Medical Academy of Continuous Professional Education, Moscow, Russia.
